# Remarkable Growth of Open Access in the Biomedical Field: Analysis of PubMed Articles from 2006 to 2010

**DOI:** 10.1371/journal.pone.0060925

**Published:** 2013-05-01

**Authors:** Keiko Kurata, Tomoko Morioka, Keiko Yokoi, Mamiko Matsubayashi

**Affiliations:** 1 School of Library and Information Science, Keio University, Tokyo, Japan; 2 Kunitachi College of Music Library, Tokyo, Japan; 3 Tokyo Institute of Technology Library, Tokyo, Japan; 4 Faculty of Library, Information and Media Studies, University of Tsukuba, Ibaraki, Japan; Johns Hopkins Bloomberg School of Public Health, United States of America

## Abstract

**Introduction:**

This study clarifies the trends observed in open access (OA) in the biomedical field between 2006 and 2010, and explores the possible explanations for the differences in OA rates revealed in recent surveys.

**Methods:**

The study consists of a main survey and two supplementary surveys. In the main survey, a manual Google search was performed to investigate whether full-text versions of articles from PubMed were freely available. Target samples were articles published in 2005, 2007, and 2009; the searches were performed a year after publication in 2006, 2008, and 2010, respectively. Using the search results, we classified the OA provision methods into seven categories. The supplementary surveys calculated the OA rate using two search functions on PubMed: “LinkOut” and “Limits.”

**Results:**

The main survey concluded that the OA rate increased significantly between 2006 and 2010: the OA rate in 2010 (50.2%) was twice that in 2006 (26.3%). Furthermore, majority of OA articles were available from OA journal (OAJ) websites, indicating that OAJs have consistently been a significant contributor to OA throughout the period. OA availability through the PubMed Central (PMC) repository also increased significantly. OA rates obtained from two supplementary surveys were lower than those found in the main survey. “LinkOut” could find only 40% of OA articles in the main survey.

**Discussion:**

OA articles in the biomedical field have more than a 50% share. OA has been achieved through OAJs. The reason why the OA rates in our surveys are different from those in recent surveys seems to be the difference in sampling methods and verification procedures.

## Introduction

### Background

The method by which efficient researchers communicate their results is essential for the development of science. Academic journals have played a significant role in scholarly communication over the past 350 years. Recently, the open access (OA) model of academic journal publishing has been the focus of considerable debate among not only publishers and librarians but also researchers, governments, and the wider public.

Although the OA model enables users to access journal articles without payment, there are many different perspectives definitions, and means of facilitating OA. The Budapest Open Access Initiative (BOAI), which articulated the public definition of OA for the first time, showed two roads toward facilitating OA worldwide: the Green and Gold roads [1]. The Green road includes tools or support to deposit peer-reviewed articles published in toll-access journals into open electronic archives such as institutional repositories (IRs) at universities. The Gold road includes researchers submitting their articles to open-access journals (OAJs) [2].

Recently, numerous efforts have been made to promote the OA model as the future model for scholarly communication. In particular, two strategies have attracted attention—the OA mandate and the mega OAJs. The OA mandate comprises strategies adopted by research funders, governments, and research institutions, requiring research output as OA [3]. The mega OAJs are a new type of OAJ, such as *PLOS ONE*. They are expected to drastically increase the availability of OA articles [4].

A decade since the BOAI, it is crucial for every stakeholder in the OA movement to assess how OA has progressed (OA trends). This article reveals OA trends in the biomedical field from 2006 to 2010, particularly the rate of OA articles per total journal articles, and the means by which articles are made freely available.

### Research questions

To determine the exact status of OA, certain studies have used the number of OAJs [5] [6] or have calculated the number of OAJ articles in few major journals in various fields. However, as Björk et al. argued [7], the most comprehensive method involves manually verifying whether articles obtained from bibliographic databases by indexing services are OA articles. Only four studies have used this method (including using a robot or automatic programming, instead of manual checking).

Hajjem et al. investigated approximately 1,370,000 records for articles published between 1992 and 2003 in 10 academic fields from the Web of Science (WoS) database. It showed the trends in OA at its start; it analyzed 660,000 biology-related articles, and concluded that the 12-year average for OA articles was 15.0% [8].

Gargoutri et al. investigated 85,215 articles published by UK academics in 14 disciplines in 2010 from the WoS, and observed an approximately 40% average across all fields, of which 35% were Green OA and 5% were Gold OA [9]. The research results of Gargoutri et al. were simply reported in a news article in *Nature*, featuring no citation of the original paper. Therefore, no details are available on the investigation method.

Björk et al. sampled articles published in 2008 from the Scopus database, and searched 1,837 articles using Google, checking the top 10 items on the first page of the search results. Across the science, technology, and medicine (STM) field, the percentage of OA articles was 20.4% on average, of which 8.5% were Gold OA and 11.9% were Green OA. In the field of medicine, OA articles recorded 21.7% on average [7].

In our previous study, which used a manual check, the OA rate was 26.0% [10].

Each of these studies used different search methods, resulting in significant variances in OA rates for the outcome. These studies investigated the rates at a single point, and none examined the change in OA rates for the same target and by the same method.

This study examines three research questions:

RQ1: To determine the progress (growth) of OA from 2006 to 2010

RQ2: To determine the most common means of making articles freely available

RQ3: To examine the factors that makes a significant difference in OA rates

## Methods

The main survey was conducted to answer RQ1 and RQ2, while supplementary surveys were conducted to answer RQ3. The sample articles for both surveys were collected from PubMed because it has been used most frequently by researchers.

### Main survey

The sample in the main survey included articles from PubMed published between January 1 and September 30 in 2005, 2007, and 2009. Thereafter, using Google, a manual check was performed to ascertain whether free full-text versions of the sample articles were available on the Web in the years following their publication, i.e., in 2006, 2008, and 2010. We named the surveys “2006 survey,” “2008 survey,” and “2010 survey,” on the basis of the year of investigation. The definite periods of each survey are shown in Table 1.

**Table 1:** Number of sample articles for each survey.

Survey year	Publication date	Accessible population	Final sample	Survey Period
2006	From January to September, 2005	9,611	4,592	From January to May, 2006
2008	From January to September, 2007	10,041	1,908	From June to August, 2008
2010	From January to September, 2009	10,859	1,942	March, 2010

#### Sampling

The sample articles were constructed by combining the year of publication with the “pagination” tag of articles in PubMed. This sampling procedure was adopted due to the practical difficulties of obtaining a random sample from the full PubMed database. More than 690,000 articles were published in 2005 at the time of the 2006 survey. PubMed did not permit the download of more than 10,000 units of bibliographic data as search results at that time. Therefore, a search query to acquire approximately 10,000 articles was initiated. Use of page numbers ranging between 11 and 19 in the “pagination” tag just resulted in a total accessible population of approximately 10,000 articles. In the 2008 and 2010 surveys, we followed the procedure of the 2006 survey.

Incidentally, editorials and other articles without authors' names or titles were eliminated. Half of the articles in 2005 and one-fifth of those in 2007 and 2009 were selected by random systematic sampling. The sample size of the 2008 and 2010 surveys was half of that of the 2006 survey. A shorter survey period is desirable because determining the OA status would change if the survey period was long. Therefore, we reduce the sample size to shorten the survey periods. Ultimately, the final samples of articles were 4,592, 1,908, and 1,942 for 2006, 2008, and 2010, respectively (see Table 1).

#### Procedure

Full-text versions of the sample articles were obtained using PubMed Central (PMC) and Google. Each database was described as follows:

1. PMC: The sample articles were searched by their PubMed ID number or titles to determine whether they were included in PMC.
2. Google: The sample articles were searched by their titles and authors' names to locate free full-text versions on Google. Moreover, only the first 20 results in the search results list were examined. When results were unobtainable by this search, we checked whether the full-text articles were provided on the journal websites.

Although Google Scholar and OAIster were also used to find the free full-text versions in the 2006 survey, the searches failed to yield results, and thus these databases were not used after the 2008 survey.

Once the articles had been located, their URLs were checked again and coded according to one of four categories: 1 = OA, 2 = restricted OA, 3 = electronic subscription journal (i.e., non-OA), and 0 = not available online. “OA” included all articles with free full-text versions available at the time of the survey, i.e., articles in OAJs, PMC, and IRs, as well as embargoed (delayed) free or sample free articles in toll-access journals. “Restricted OA” included articles for which users have to register to gain access, and articles that contain only text (no figures and tables) freely available online. “Electronic subscription journal” included all articles that required a subscription to the journal or for which the reader had to pay on a pay-per-view basis to access them. “Not available online” included articles without a full-text version available on the Web.

### Supplementary surveys

The OA rate is calculated easily using two functions provided by PubMed: a “LinkOut” search or “Limits” (currently named “filter”) search. Both functions have been available on PubMed for several years. The rates of OA articles have been consistently calculated by these two methods.

The main survey sample articles were re-searched using the LinkOut functionality in PubMed. The LinkOut service provides links to full-text articles based on publishers' information. There are three links: “full-text,” “free full-text,” and “free full-text in PubMed Central (currently PMC).” Articles in the search results that had “free full-text” or “PubMed Central (currently PMC)” icons were considered as OA articles. We used this procedure to calculate the OA rates from 2008 to 2011.

A Limits search can narrow the search results by “text availability”: “full-text,” “free full-text,” and “has abstract.” The articles published in each year were re-searched using the “free full-text” link. We considered the figure obtained by this procedure as the OA rate. The OA rate for each year was calculated for 2007 to 2011.

## Results

### Rising percentage of OA articles

**Figure 1:**
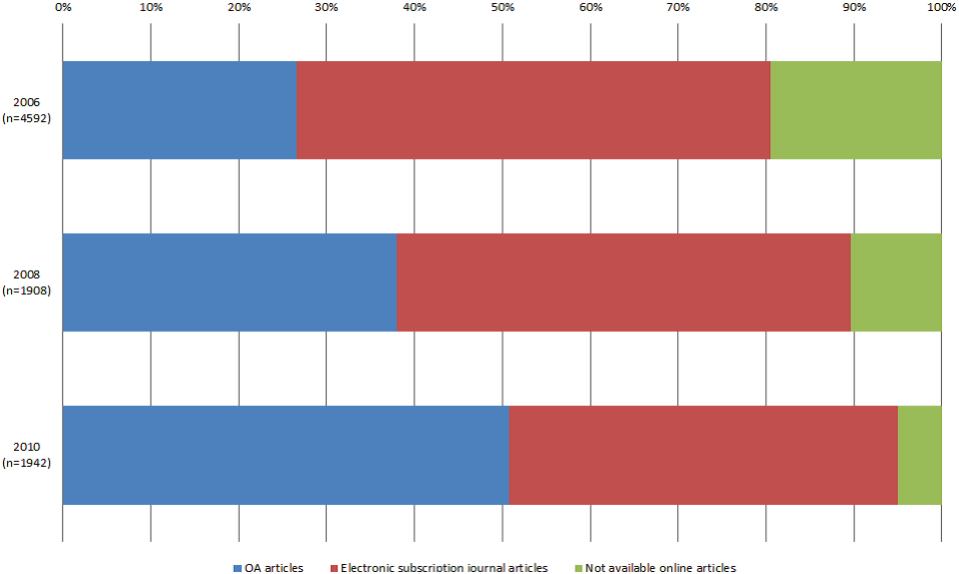
Rising percentage of OA.

Figure 1 shows the rates of articles under the “OA,” “electronic subscription journal,” and “not available online” categories for 2006, 2008, and 2010. “Restricted OA” was excluded from Figure 1 because of negligible percentages across the years and was, therefore, not considered as a significant influence in overall OA trends. The OA articles were counted once, irrespective of availability in multiple locations.

The rate of OA articles increased significantly from 2006 to 2010. In the 2010 survey, half of the sample articles (50.2%) were OA articles, which was twice the percentage of those in the 2006 survey. “Electronic subscription journals” consistently comprised approximately half of the sample articles for all three years (53.5%, 50.7%, and 43.8% for 2006, 2008, and 2010, respectively), although they decreased marginally in 2008 and 2010. In 2010, the rate of OA articles exceeded that of electronic subscription journal articles for the first time. Moreover, the rate of articles listed as “not available online” declined significantly between 2006 and 2010 (19.3%, 10.1%, and 5.0% for 2006, 2008, and 2010, respectively), indicating that most of the articles in the biomedical field that are indexed in PubMed are available online.

### Transition in the method for providing OA

The task of determining whether sample articles were OA was treated singularly in the surveys, i.e., OA articles were only counted once, irrespective of their availability on multiple OA websites. For example, it was possible to find an OA article simultaneously in an OAJ in PMC and an IR. Because, in our opinion, this duplicative status is an indicator of the progressive expansion of OA, we did not focus solely on one method of OA provision, but instead counted all available methods repeatedly. Therefore, the total percentages of OA provision inevitably totaled more than 100% in each survey.

Our surveys' results showed a dynamic and progressive expansion of OA in 2006, 2008, and 2010. For each survey, the OA articles were classified according to the OA provision methods available at that time. A new set of OA provision categories was developed, and the article samples were recalculated to compare the three original surveys. These categories are as follows:

1. OAJ
2. Toll-access journals
3. PMC
4. IRs and websites of institutions
5. Personal websites
6. Free article databases
7. Others

**Figure 2:**
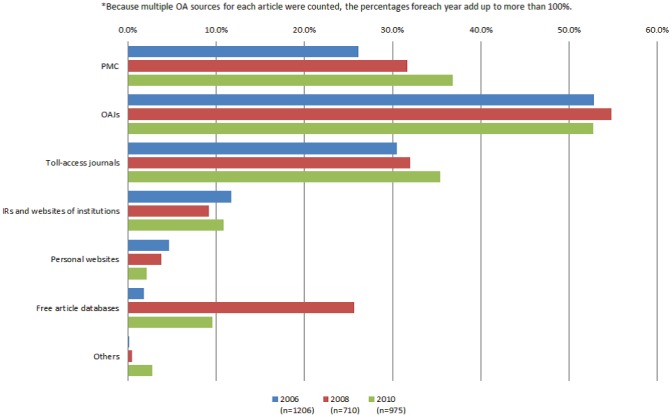
Transitionin OA provision methods.^*^ * Because multiple OA sources for each article were counted, the percentages for each year add up to more than 100%.

A comparison of the aggregate results obtained from the three surveys is shown in Figure 2. As noted earlier, the total percentages are greater than 100% because of multiple counts for the methods.

In each survey, the majority (52.8%, 54.8%, and 52.7% for 2006, 2008, and 2010, respectively) of OA articles were available from OAJ websites, indicating that OAJs have consistently been a significant contributor to OA. Conversely, the rate of OA articles available from PMC also increased significantly (from 26.1% in 2006 to 36.8% in 2010).

In contrast, the rates of OA articles available from IRs or websites was approximately 10%, while those of OA articles available from authors' websites remained under 5% in each survey. Therefore, we can infer that IRs and institutional and authors' websites have significantly less influence on the OA status as compared with other methods. Furthermore, the rates of OA articles available from free article databases were rather inconsistent (1.8%, 25.6%, and 9.5% for 2006, 2008, and 2010, respectively). Although the reason for the significant decline in the rate of OA articles from 2008 to 2010 from such databases is still unclear, the fluctuating rates indicate that free article databases cannot be a significant, stable method for providing OA.

The percentage of OA articles that were available through multiple ways in the 2010 survey was 40.1% (391 articles), while 59.9% of those were available only on a single platform. The most frequently appearing pattern was articles being available from PMC and OAJ simultaneously. Other typical patterns were articles being available from PMC, OAJ, and free article databases, or from PMC and toll-access journals as samples or embargoed articles.

### The OA rate obtained by Alternative methods

Table 2 presents the results of the two supplementary surveys in which OA rates were calculated using PubMed's LinkOut and Limits functionalities. Table 2 also presents the results of the main survey to facilitate a comparison between the supplementary and the main surveys.

**Table 2:** Comparison of OA rates obtained by supplementary surveys and the main survey.

Survey year		2006	2007	2008	2009	2010	2011
Publication year		2005	2006	2007	2008	2009	2010
PubMed: LinkOut	Number of articles	-	-	1,963	1,977	1,942	1,874
	Number of OA Articles	-	-	342	428	474	531
	OA rate	-	-	17.4%	21.6%	24.4%	28.3%
PubMed: Limits	Total number of articles in PubMed	-	731,576	765,043	813,867	854,111	925,047
	Number of articles with free full-texts	-	144,156	163,450	188,080	215,703	248,520
	OA rate	-	19.7%	21.4%	23.1%	25.3%	26.9%
Main Survey	Number of articles	4,592	-	1,908	-	1,942	-
	OA articles	1,248	-	747	-	995	-
	OA rate	27.2%	-	39.2%	-	51.2%	-

PRF: Pls confirm that vertically merged cells and diagonal lines are correctly formatted.

The results of the LinkOut survey show that the rate of OA articles has been gradually increasing from 17.4% in 2008 to 28.3% in 2011. However, there is a significant difference between the OA rates in the LinkOut survey and the main survey, although the sample articles were selected by the same method. This difference could be due to the OA percentage between our main survey and previous studies.

In the Limits survey, the OA percentage gradually increased from 19.7% in 2007 to 26.9% in 2011. Although the OA percentages obtained from the Limits survey are marginally higher than those obtained from the LinkOut survey, they still comprise approximately half the percentages in the main survey.

In a comparison between our Google search in the main survey and the PubMed LinkOut survey, 60% of articles for which there was no information in the LinkOut survey corresponded to OA articles in the main survey. It is likely that LinkOut relies on the information provided to PubMed by larger publishers, and therefore may have less information for journals published by smaller publishers or publishers from non-Anglophone countries.

## Discussion

For RQ1, the results of the main surveys in 2006, 2008, and 2010 indicate significant progress in the status of OA in the biomedical field in the last five years. It is noteworthy that the percentage of OA in the 2010 survey is above 50%, which is considerably larger than the OA availability reported in previous studies.

For RQ2, OAJs have consistently made the most significant contribution to OA expansion during our survey period. The availability of OA articles from PMC has been increasing steadily, but the number of articles that have been made available directly by authors has remained low (0.2%, 3.0%, and 4.0% for 2006, 2008, and 2010, respectively). The expansion of PMC article availability has been achieved, not by individually providing the “authors' manuscripts,” but by making entire journal issues available on OA through PMC (including OAJs and embargoed articles from toll-access journals). PMC serves both as a repository for Green OA articles as part of the Public Access Policy and also an electronic journal platform for Gold OA articles provided by journal publishers.

In addition, OA articles available through multiple ways were 40.0% in 2010, which was higher than the 11% recorded in Way's survey of OA articles in the field of library and information science [11]. Therefore, it can be argued that relatively high levels of plurality in OA sources may be a characteristic of the biomedical field—the two definitions of OA in BOAI: the Green and Gold roads were ineffective, because OA provision methods have become diverse and complex.

For RQ3, the main factors (reasons) for the disparity in the results of OA rates between our research and similar studies may be as follows: (1) differences between bibliographic databases from which target articles were extracted; (2) detailed sampling methods; and (3) determining (checking) the verification procedure for OA articles. Many studies, especially at the beginning of the OA movement, used WoS [12], which includes 12,000 prestigious refereed journals from all fields in the humanities, social sciences, and natural sciences. Recently, Björk et al. used Scopus, which contains about 20,000 refereed journals across all fields (in 2012). It also has 6,000 journals in health science, and has “a 100% overlap with Medline titles.” [13]

This study, however, used PubMed. “PubMed is the most widely used tool for searching biomedical and life science literature online. During fiscal year 2011 there were about three to six million user queries to PubMed each day” [14]. In addition, according to a 2007 survey, approximately 90% of Japanese medical researchers used PubMed at least once a week [15]. PubMed primarily comprises three articles: 1) articles indexed in the MEDLINE database; 2) OAJs included in PMC; and 3) “author manuscripts,” submitted according to NIH Public Access Policy [16]. The number of journals included in PubMed is not publicly available; however, MEDLINE, which comprises majority of PubMed, indexed about 5,600 journals [17].

WoS certainly reflects the trends of prestigious journals. Although both Scopus and PubMed include all articles from the MEDLINE database, there is a possibility that PubMed may include more OA articles than Scopus due to the OAJs and OA articles included in PMC.

As for the detailed sampling procedure, there were no studies that used random sampling for manually checking OA articles. Both our study and that of Björk et al. did not use random sampling. Our survey has the possibility of bias toward small journals due to the lower number of pages used for sampling.

Finally, although both the main and supplementary surveys targeted the same database, PubMed, the OA rates recorded in both surveys were significantly different. This distinction showed that determining (checking) the verification procedure for OA articles has any effect on OA rates. Our manual checking used in the main survey was comprehensive, and we developed expertise in effectively searching OA articles in the biomedical field. Therefore, we could effectively determine OA articles, of which were not judged OA using PubMed's LinkOut functionality.
